# A Patient with a Right Atrium Mass and Congenital Heart Disease: A Challenging Diagnosis of a Stubborn Disease

**DOI:** 10.1155/2016/6810961

**Published:** 2016-08-21

**Authors:** Wenyan Wang, Huaicong Long, Zhiying Zhao

**Affiliations:** ^1^Heart Failure Center, Sichuan Provincial People's Hospital, Chengdu, Sichuan 610072, China; ^2^Geriatric ICU, Sichuan Provincial People's Hospital, Chengdu, Sichuan 610072, China

## Abstract

Cardiac lymphoma is extremely rare. An intracardiac mass has rarely been reported to be the cardiac involvement of extranodal lymphoma. It is difficult to establish a final diagnosis via routine examinations. The ability of an echocardiogram to characterize tissue is limited; systemic (18)F-FDG PET/CT scans provide important information for both staging and response assessment in patients with lymphoma. A 68-year-old Chinese male with a second patent foramen ovale (PFO) and an interventricular septal defect presented at our institute with persistent fever, shortness of breath, repeated paroxysmal supraventricular tachycardia (PSVT) attack, and rapidly progressing superior vena cava syndrome. The patient also presented with a mass located in the upper right atrium and superior vena cava which was detected by echocardiogram. (18)F-FDG PET/CT scan revealed a pathological increase of (18)F-FDG uptake in the atrial mass and several other extracardiac lymph nodes. Lymph node biopsy was positive for large B-cell lymphoma. Immunohistochemistry revealed intense and diffuse expression of CD20, CD10, BCL-6, and Ki-67. The patient died without any chemotherapy 18 days after hospital discharge.

## 1. Introduction

Cardiac masses arising from the heart or the pericardium are rare [1] and potentially lethal. Cardiac Non-Hodgkin's lymphoma (NHL) is extremely rare and accounts for 1-2% of primary cardiac tumors involving the right versus left atrium at a ratio of approximately 8 : 1 [2, 3]. Older patients with untreated congenital heart disease and the comorbidity of cardiac lymphoma are even less common and have a poor prognosis. We report the case of an old man who had initially presented with PFO/VSD heart failure and endocarditis and then was later diagnosed with diffuse large B-cell lymphoma (DLBCL) in his right atrium after a mass was discovered via PET/CT.

## 2. Case Report

A 68-year-old Chinese male presented to the geriatric ICU in May, 2015, with a “persistent fever lasting two months and worsening shortness of breath lasting one month.” Two months prior to presenting to the ICU, the patient began to experience shivering and hyperpyrexia reaching 40°C and a cough with phlegm production. He denied chest pain and hemoptysis. One month prior to presenting to the ICU, he began to complain of palpitations and edema in his lower extremities. He received antibiotics and diuretics without any progress. Although he was previously diagnosed with chronic bronchitis and pulmonary emphysema many years earlier, PFO and VSD remained untreated. He has been a smoker for 40 years and there is nothing significant in his family history.

Vital signs at time of admission were as follows: temperature 38.8°C, pulse 150 bpm, being conscious and anicteric, no cyanosis, and an operative lymph node palpable in the left iliac region without tenderness. Coarse crackles were heard at the base of lungs bilaterally, as well as cardiac murmurs at the third and fourth intercostal space on the left bounder of the sternum. Moderate pitting edema of both lower extremities was also observed. Laboratory tests were WBC 7.37 × 10E9/l, N 75.8%, Hb 105 g/L, C-reactive protein (CRP) 89.15 mg/L, BNP 523 pg/mL, PCT 0.25, and ESR 95 mm/h. Renal function and liver enzymes were within normal limits. Blood plasmodium test and ANA spectrum were negative. Blood culture was* Staphylococcus hominis* (sensitive to vancomycin). ECG was paroxysmal supraventricular tachycardia (PSVT) with HR at 155 beats/min. Chest X-ray showed there was some patchy consolidation at the right upper lung and bilateral pleural effusion. The lymph nodes with reactive hyperplasia were detected by ultrasound at bilateral inguinal regions.

**Figure 1:**
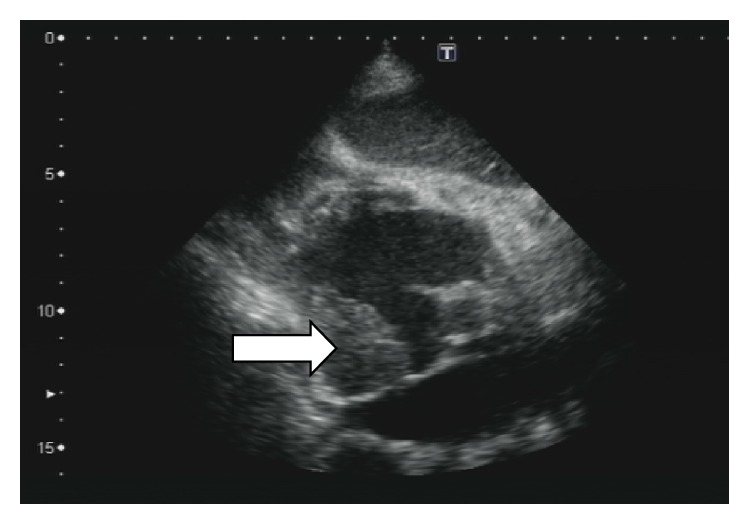
Upper right atrium and superior vena cava mass. Subcostal view of dual atrium demonstrated a lobulated and echo reflectant mass (arrow) in upper right atrium and superior vena cava.

**Figure 2:**
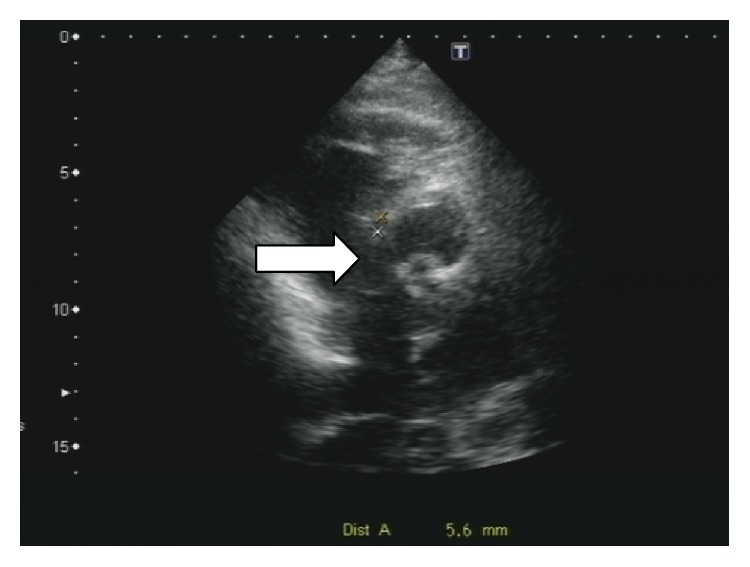
Ventricular septal defect (VSD). Parasternal short view showed the VSD (arrow).

Transthoracic echocardiography (TTE) is shown in Figures 1-2.

**Figure 3:**
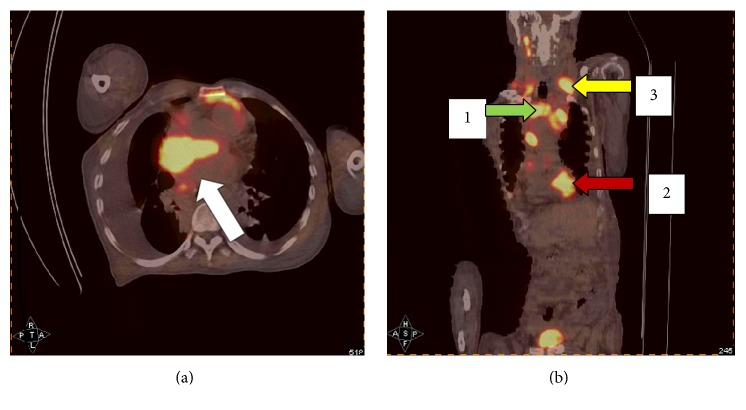
Systemic (18)F-FDG PET/CT scan revealed that the (18)F-FDG uptake in the mass located in the right atria pathologically increased ((a) arrow). Quite a few lymph nodes with increased uptake of (18)F-FDG were revealed at the regions of neck (yellow arrow), mediastinum (green arrow), and right atrium (red arrow) (b).

After his admission, the patient was put on intravenous cefoperazone-tazobactam 2.5 g q12h for 16 days and intravenous Vancomycin 500 thousand q8h for 5 days and then increased to q6h for 11 days. The patient was also put on Propafenone, metoprolol, Perindopril, diuretics, and glyceryl trinitrate. He continued to experience worsening shortness of breath and orthopnea and swelling in his right upper limb, and ECG telemonitoring showed repeated attack of PSVT. The patient was suspected to have developed superior vena cava syndrome; furthermore, the patient received a vascular ultrasound with no thrombosis detected, and BNP reduced to 159 ng/mL. Systemic (18)F-FDG PET/CT scan (Figures 3(a) and 3(b)) revealed that the (18)F-FDG uptake in the mass located in the right atria pathologically increased. The patient finally received lymph node biopsy: large B-cell lymphoma, CD20(+) CD3 T cell(+) Bcl-6(+), CK(+) CD21(−) CD10(−) MUM-1(−) EBER(−). The patient and his family declined chemotherapy and he was discharged from the hospital. He died after 18 days of following up.

## 3. Discussion

The incidence of NHL has increased in recent decades in Asia [4]. But lymphomas rarely involve the heart with incidence between 9% and 24% on autopsy series. NHL can affect the heart but usually in the form of pericardial effusion which is often associated with direct epicardial spread [5]. Only 0.5% of all extranodal lymphomas present as cardiac neoplasms [6].

Cardiac masses arising in the heart or the pericardium are potentially lethal; they have variable symptoms which lack specificity; the clinical presentation is determined utilizing numerous factors. Depending on tumor location, size, growth rate, degree of invasion, and friability, the clinical presentations could include fever, fatigue, arthralgia, refractory congestive heart failure or arrhythmia, cardiomegaly, pericardial effusion, sudden death [7], and superior vena cava syndrome. The rarity and heterogeneous clinical presentation of PCL make its diagnosis difficult. Imaging studies such as TEE may not be reliable in distinguishing cardiac lymphoma from other cardiac neoplasms including myxomas, angiosarcoma, or rhabdomyomas. Endomyocardial biopsy, excisional intraoperative biopsy, and pericardial fluid cytological evaluation should be utilized to establish final diagnosis.

We are reporting this case of NHL and massive intracardiac involvement diagnosed by echocardiographic and PET/CT assessment. The patient's heart defect, medical history of lung disease, persistent fever, and positive blood culture are what guided the original misdiagnosis of hematosepsis and endocarditis. It is hard to explain how come even BNP decreased but refractory arrhythmia and superior vena cava syndrome were getting worsen based on aggressive treatment of antibiotics, diuretics, and vassal dilator. Although TTE is the preferred initial imaging technique used in the detection and diagnosis of cardiac masses, its ability to characterize tissue is limited. (18)F-FDG PET/CT scanning is superior in this regard and, due to its noninvasive approach, should therefore be the diagnostic tool of choice for cardiac mass assessment for the elderly, particularly those who might lose the chance to receive invasive procedures such as TEE or myocardial biopsy. The sensitivity of FDG PET/CT imaging was reported to be 71–100%, with a specificity of 69–100% and a negative predictive value of 80–100% [8, 9].

The prognosis for patients with cardiac sarcomas remains very poor [10]. DLBCL is an aggressive lymphoma that is typically treated with chemotherapy and radiation therapy which could improve the survival rates [11]. Early systemic chemotherapy, in addition to restricting the tumor, might yield a better prognosis.
